# A sustained deficiency of mitochondrial respiratory complex III induces an apoptotic cell death through the p53-mediated inhibition of pro-survival activities of the activating transcription factor 4

**DOI:** 10.1038/cddis.2014.469

**Published:** 2014-11-06

**Authors:** A G Evstafieva, A A Garaeva, A A Khutornenko, A V Klepikova, M D Logacheva, A A Penin, G E Novakovsky, I E Kovaleva, P M Chumakov

**Affiliations:** 1 Belozersky Institute of Physico-Chemical Biology, Lomonosov Moscow State University, Moscow, 119992, Russia; 2 Faculty of Bioengineering and Bioinformatics, Lomonosov Moscow State University, Moscow, 119992, Russia; 3 Faculty of Biology, Department of Genetics, Lomonosov Moscow State University, Moscow, 119992, Russia; 4 Engelhardt Institute of Molecular Biology, Russian Academy of Sciences, Vavilova Street 32, Moscow, 119991, Russia

## Abstract

Generation of energy in mitochondria is subjected to physiological regulation at many levels, and its malfunction may result in mitochondrial diseases. Mitochondrial dysfunction is associated with different environmental influences or certain genetic conditions, and can be artificially induced by inhibitors acting at different steps of the mitochondrial electron transport chain (ETC). We found that a short-term (5 h) inhibition of ETC complex III with myxothiazol results in the phosphorylation of translation initiation factor eIF2*α* and upregulation of mRNA for the activating transcription factor 4 (ATF4) and several ATF4-regulated genes. The changes are characteristic for the adaptive integrated stress response (ISR), which is known to be triggered by unfolded proteins, nutrient and metabolic deficiency, and mitochondrial dysfunctions. However, after a prolonged incubation with myxothiazol (13–17 h), levels of ATF4 mRNA and ATF4-regulated transcripts were found substantially suppressed. The suppression was dependent on the p53 response, which is triggered by the impairment of the complex III-dependent *de novo* biosynthesis of pyrimidines by mitochondrial dihydroorotate dehydrogenase. The initial adaptive induction of ATF4/ISR acted to promote viability of cells by attenuating apoptosis. In contrast, the induction of p53 upon a sustained inhibition of ETC complex III produced a pro-apoptotic effect, which was additionally stimulated by the p53-mediated abrogation of the pro-survival activities of the ISR. Interestingly, a sustained inhibition of ETC complex I by piericidine did not induce the p53 response and stably maintained the pro-survival activation of ATF4/ISR. We conclude that a downregulation of mitochondrial ETC generally induces adaptive pro-survival responses, which are specifically abrogated by the suicidal p53 response triggered by the genetic risks of the pyrimidine nucleotide deficiency.

Mutations in the mitochondrial genome or in the nuclear genes related to mitochondrial functions are associated with a wide set of mitochondrial diseases that share some common changes in transcriptome.^1, 2^ In particular, there are evidences for common induction of the unfolded protein response (UPR)- or the integrated stress response (ISR)-associated genes, including activating transcription factor 4 (ATF4) and its target genes, C/EBP homologous protein (CHOP) and asparagine synthetase (ASNS).^2, 3^ Mitochondrial dysfunction induced by an inhibition of mitochondrial electron transfer chain (ETC) complex I with rotenone was also shown to induce the expression of the UPR/ISR genes ATF4 and CHOP.^4^

Environmental stresses induce rapid changes in gene expression that eventually alleviate cell damage and return cells to homeostasis. Different environmental stresses induce the phosphorylation of translation initiation factor eIF2*α* at Ser 51 by protein kinases PERK (ER stress), GCN2 (nutrient depletion), PKR (viral infection) or HRI (heme deprivation), resulting in the global repression of protein biosynthesis^5^ that promotes viability of cells during mitochondrial dysfunction.^6^ In addition to the global attenuation of translation, eIF2*α* phosphorylation leads to an increased translation of mRNAs with small upstream open reading frames, including the transcription factor ATF4.^5^ ATF4 is a transcriptional activator of the genes involved in nutrient uptake, metabolism, redox regulation and apoptosis. ATF4 acts as a common downstream target that integrates signals from different eIF2 kinases, and therefore the eIF2*α*/ATF4 pathway is commonly dubbed as ISR.

In addition to the translational control, the ATF4 expression is subjected to a transcriptional regulation. Some stress conditions, such as exposure to UV irradiation, do not increase the ATF4 protein expression despite a robust eIF2*α* phosphorylation.^7^ Under these conditions, the *ATF4* gene is deeply repressed and the ATF4 mRNA is not available for the preferred translation. The combination of transcriptional and translational regulation allows the eIF2 kinase pathway to selectively control key regulatory genes subjected to preferential translation, thereby contributing to the balance between stress remediation and apoptosis.^7^

Here, we found that an inhibition of mitochondrial complex I with piericidine results in a time-dependent increase in the ATF4 mRNA expression levels. A similar increase was observed during a short-term inhibition of complex III with myxothiazol; however, there was a deep repression of ATF4 transcription during the sustained treatment with the drug. We have shown previously that inhibition of mitochondrial ETC specifically within complex III results in an activation of the p53 tumor suppressor because of an impairment of the *de novo* pyrimidine biosynthesis.^8^ We show that the activation of p53 can modify the ISR induced by mitochondrial dysfunction. After a short exposure to myxothiazol, we detected phosphorylation of eIF2*α* suggesting the induction of the eIF2*α*/ATF4 pathway. However, a substantial inhibition of the pathway was observed after a long-term complex III inhibition because of the downregulation of *ATF4* mRNA. By following transcriptome changes in response to complex III inhibition, we reveal a cross-talk between p53 and ATF4, which decides the fate of the affected cell.

## Results

### Differential expression of ATF4 and its target genes after mitochondrial ETC complex III inhibition

To study the response of cells to stress induced by inhibition of the mitochondrial ETC complex III, we monitored by mRNA-seq the transcriptome changes following myxothiazol treatment. We used the gene ontology analysis tool DAVID^9^ to assess the enrichment of transcripts corresponding to functional groups within the list of differentially expressed genes relative to their representation within the genome. After 5 h of myxothiazol treatment, the upregulated transcripts were substantially enriched with those involved in translation (FDR 3.09E-20) and the ribosome pathway (FDR 7.4E-18). According to the ChIP-seq data,^10^ at this point, the most significantly enriched biological functions correspond to those of the genes controlled by ATF4. However, after 13–17 h of myxothiazol treatment, the most enriched functions corresponded to the p53 pathway (FDR 1.23E-06).

As it has been reported that the ISR genes ATF4, CHOP and ASNS are upregulated with significant probability in response to a mitochondrial dysfunction,^2, 3, 4^ we tested the expression of ATF4 and its target genes following inhibition of complex III. Indeed, the ATF4 and several known ATF4-dependent genes (encoding ASNS, transcription regulators CHOP and TRIB3, amino acid transporter SLC7A11 and cation transport regulator homolog CHAC1) were identified among the genes upregulated as early as after 5 h of myxothiazol treatment (Table 1), suggesting the induction of the ISR/UPR pathway.

**Figure 1:**
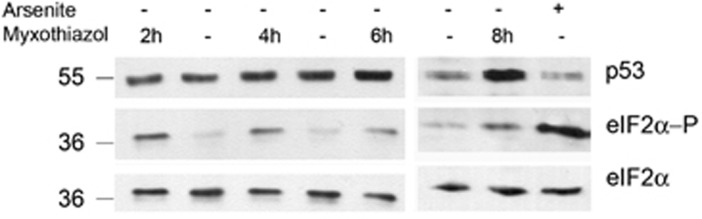
Western analysis of p53, phosphorylated eIF2*α* (eIF2*α*-P) and eIF2*α* in myxothiazol- or arsenite-treated RKO cells for indicated intervals of time

To assess the contribution of the eIF2*α*/ATF4 pathway in the cellular response to complex III inhibition, we measured phosphorylation at Ser51 of translation initiation factor eIF2*α* in cells treated with myxothiazol. The phosphorylated eIF2*α* was detected after 2 h exposure to myxothiazol and remained at least until 8 h (Figure 1). Levels of eIF2*α* phosphorylation were substantial, but lower than that in the cells treated with arsenite, an inducer of UPR/ISR used as a positive control. As eIF2*α* phosphorylation usually leads to an increased translation of ATF4 mRNA, the data suggest that the ISR/ATF4 pathway may contribute to the response induced by the dysfunctional ETC complex III.

**Figure 2:**
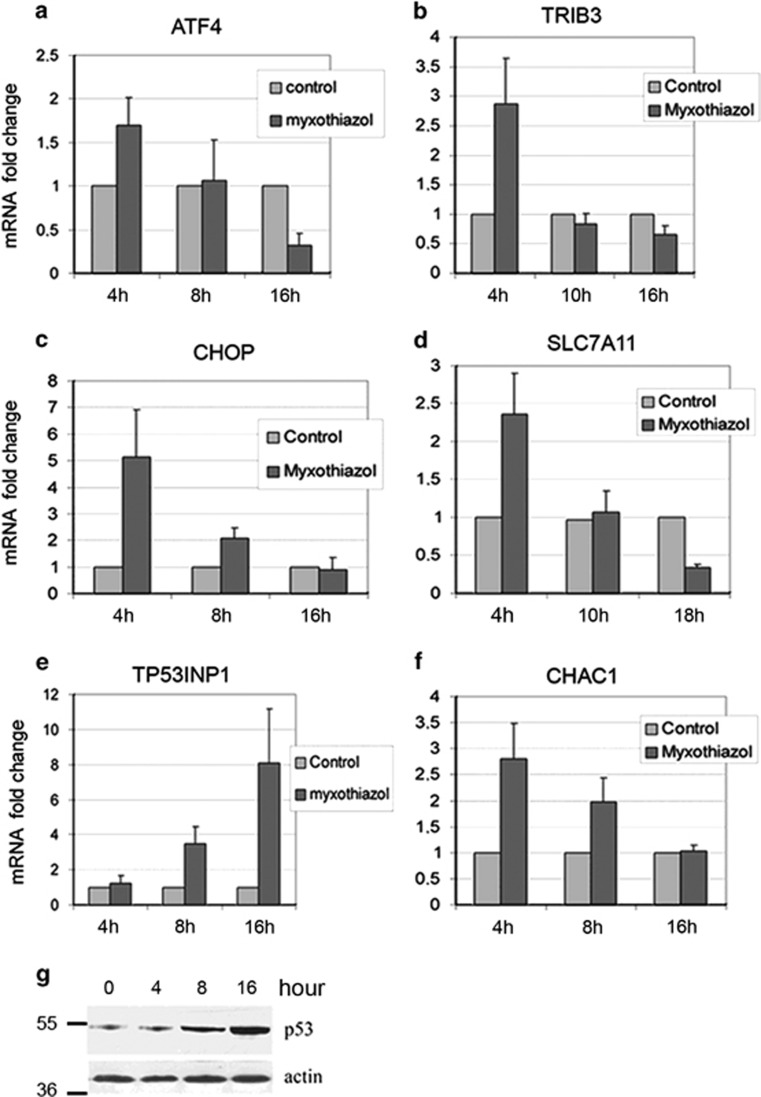
The time-dependent effect of mitochondrial ETC complex III inhibition on the expression of ATF4 and its target genes in HCT116 cells. (**a–f**) The effects of myxothiazol (1 *μ*M) for indicated intervals of time on ATF4, CHOP, TRIB3, SLC7A11, CHAC1 and TP53INP1 mRNA levels in HCT116 cells were examined by RT-qPCR. Mean and S.D. are presented of three independent experiments. All values are normalized to the level of the corresponding mRNA in the control (untreated) cells. (**g**) Western analysis of p53 in myxothiazol-treated HCT116 cells for indicated intervals of time

Surprisingly, at the later time points of complex III inhibition (13–17 h), the expression of ATF4 mRNA switched from a 2.5-fold upregulation to a 2.3- and 4-fold downregulation (Table 1). The expression of the above ATF4 target genes (except ASNS) either returned to control levels (CHOP) or even dropped further below (CHAC1, SLC7A11, TRIB3). After 17 h of myxothiazol treatment, the upregulated ASNS mRNA levels also decreased from 3.6-fold at 5 h to 1.5-fold. Apparently, the changes observed following ETC inhibition could not be explained simply by the UPR/ISR gene expression program. To clarify this point, the effect of complex III inhibition on ATF4 mRNA levels at different time points was examined by RT-qPCR analysis. We observed a time-dependent change in ATF4 mRNA level in HCT116 (Figure 2a), RKO (Supplementary Figure S1a) and HeLa (Supplementary Figure S2a) cell lines. Following myxothiazol treatment, the levels of ATF4 mRNA were increased at an early time point (4 h) and then dropped below the control level (16 h). A similar time dependence (but sometimes shifted toward later time points) was obtained for the selected ATF4 transcriptional targets DDIT3/CHOP, TRIB3, SLC7A11 and CHAC1 (Figures 2 b–d, f; Supplementary Figure S1 b–d, f; Supplementary Figure S2 b–d). Therefore, the RT-qPCR analysis has confirmed the mRNA- seq results and has shown that the suppression of ATF4 in response to a sustained ETC complex III inhibition was not cell-line specific. Besides, western analysis has shown that following myxothiazol treatment, the ATF4 protein levels were also increased at early time points (3–5 h) and then dropped to (16 h) or below (24 h) the control levels (Supplementary Figure S1h).

Meanwhile, the accumulation and activation of p53 started after 8 h of myxothiazol treatment, as was evidenced by western analysis and by RT-qPCR data showing elevated expression of the p53-responsive gene TP53INP1 (Figure 2e; Supplementary Figure S1e and g). Note that there were reciprocal relations between the accumulation of mRNA of ATF4 or of its target genes and the accumulation of TP53INP1 mRNA suggesting interference between ATF4 and p53 activities during respiration chain complex III inhibition.

To identify which of the potential targets of ATF4 were actually induced in an ATF4-dependent manner in response to a short-term complex III inhibition, we stably expressed ATF4-specific short hairpin RNAs (shRNAs) in RKO cells where an induction of potential ATF4 target genes was more pronounced. As shown in Supplementary Figure S3, we were able to decrease the myxothiazol-induced expression of ATF4 mRNA by >80% using two separate shRNA targeting its different regions of ATF4 mRNA, compared with a scrambled shRNA control. Next, we examined the expression of ATF4-regulated genes in RKO cells treated with myxothiazol for 4 h. There was a significant suppression of the myxothiazol-induced expression of TRIB3, ASNS and SLC7A11 (but not DDIT3/CHOP) in the ATF4 knockdown sublines (Supplementary Figure S3). These data indicate that the induction of TRIB3, ASNS and SLC7A11 expression in response to a short exposure to myxothiazol depends strongly on ATF4. The above three ATF4 target genes were selected for further work.

**Figure 3:**
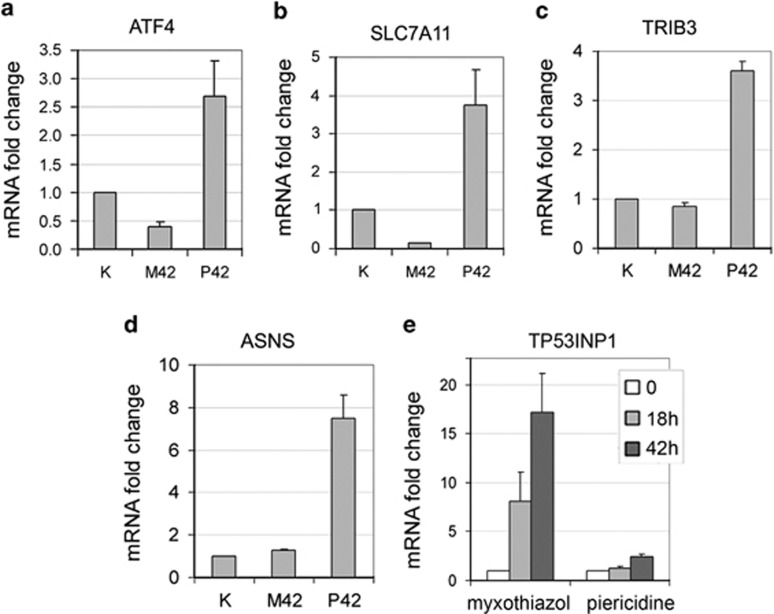
In contrast to complex III inhibition by myxothiazol, complex I inhibition by piericidine does not result in p53 activation, and leads to long-term enhanced expression of ATF4 and its target genes. (**a**–**d**) The effects of 1 *μ*M myxothiazol (M42) and 2 *μ*M piericidine (P42) treatment for 42 h on ATF4, TRIB3, ASNS and SLC7A11 mRNA levels in RKO cells were examined by RT-qPCR. All values are normalized to the level of the corresponding mRNA in the control (untreated) cells. Mean and S.D. are presented of three independent experiments. (**e**) The effects of myxothiazol (1 *μ*M) and piericidine (2 *μ*M) treatment for 18 h and 42 h on TP53INP1mRNA levels in RKO cells were examined by RT-qPCR. Mean and S.D. are presented of three independent experiments. All values are normalized to the level of the corresponding mRNA in the control (untreated) cells

We found that, unlike the complex III inhibitor myxothiazol, a complex I inhibitor piericidine did not induce an activation p53, but it led to a sustained upregulation of ATF4 and its target genes (Figure 3), suggesting that the specific induction of p53 by myxothiazol is responsible for the switch of the ATF4-dependent gene expression from an upregulation to its suppression.

### Abolishment of p53 activation by uridine supplementation prevents the ATF4 mRNA downregulation in response to a prolonged complex III inhibition

**Figure 4:**
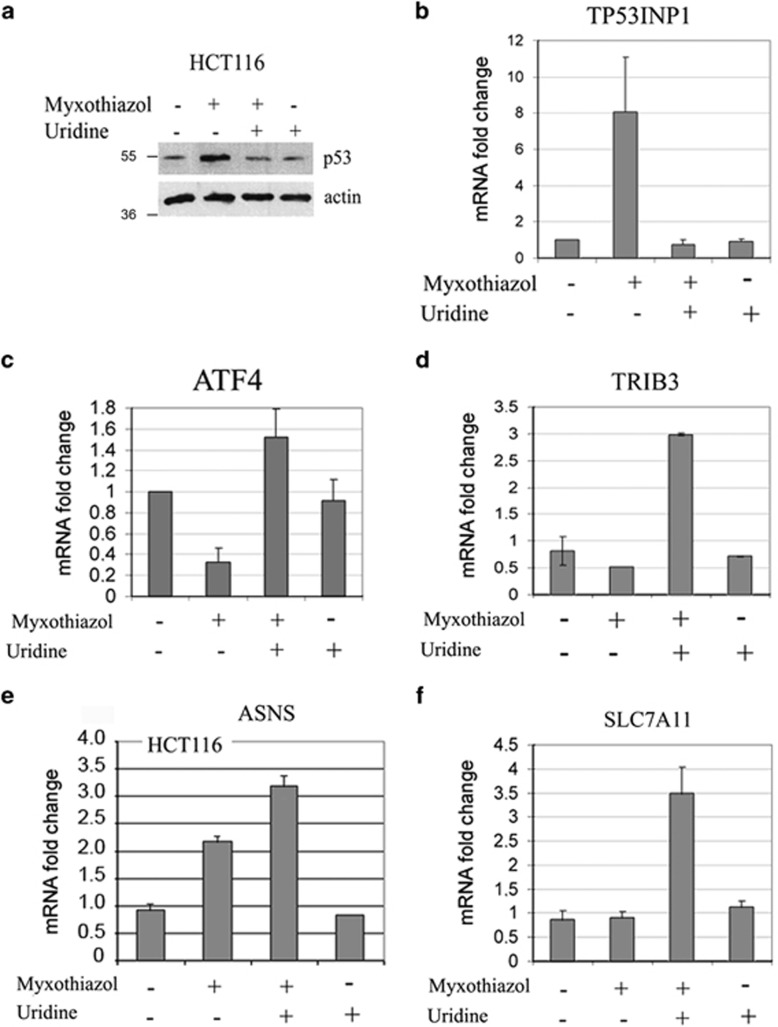
Abolishment of p53 activation by uridine supplementation prevents the downregulation of ATF4 in response to complex III inhibition and stimulates the expression of ATF4 target genes. (**a**) p53 accumulation was analyzed by western analysis in HCT116 cells treated for 13 h with myxothiazol or/and uridine as indicated. (**b**–**f**) TP53INT1, ATF4, TRIB3, ASNS and SLC7A11 mRNA levels in HCT116 cells treated in the same way were examined by RT-qPCR. Mean and S.D. are presented of three independent experiments. All values are normalized to the level of the corresponding mRNA in the control (untreated) cells

As the induction of p53 coincided with the downregulation of ATF4, we checked whether a prevention of the p53 activation could restore the elevated ATF4 expression. As the inhibition of complex III induces activation of p53 because of the impairment of pyrimidine biosynthesis,^8^ we tested whether a replenishment of pyrimidine nucleotide pools abrogates the myxothiazol-induced inhibition of ATF4 expression. The mRNA-seq and RT-qPCR analyses were performed after treatment of HCT116 cells with myxothiazol in the presence of uridine, which is a precursor of uridylic and cytidylic nucleotides. Indeed, the co-supplementation of HCT116 cells with uridine completely abolished the myxothiazol-induced accumulation of p53 (Figure 4a) and the elevated expression of the p53 target gene TP53INP1 (Figure 4b). The functional clustering among the upregulated genes after treatment with myxothiazol plus uridine for 13 h has shown an enrichment with transcripts encoding proteins involved in translation (FDR 0.0025) and the ribosome pathway (FDR 0.05), as it was observed after myxothiazol treatment for 5 h. Also, in the presence of uridine, there was no enrichment with transcripts involved in the p53 signaling pathway. The mRNA-Seq (Table 1) and RT-qPCR data (Figure 4c, Supplementary Figure S4c) indicate that the supplementation of HCT116 or RKO cells with uridine largely reversed the ATF4 mRNA downregulation after prolonged exposure to myxothiazol. When the cells were treated with myxothiazol in the presence of uridine, there was also an upregulation of the ATF4 target genes TRIB3, SLC7A11 and ASNS (Figure 4d and f,Supplementary Figure S4 d–f). Besides, the uridine supplementation without myxothiazol did not affect mRNA levels for ATF4 and its downstream targets (Figure 4). Collectively, the results indicate that although uridine itself does not stimulate the expression of ATF4, it blocks the activation of p53 and prevents the ATF4 mRNA downregulation in response to a sustained inhibition of complex III.

### Preactivation of p53 prevents the ATF4 upregulation in response to a short-term inhibition of respiration chain

**Figure 5:**
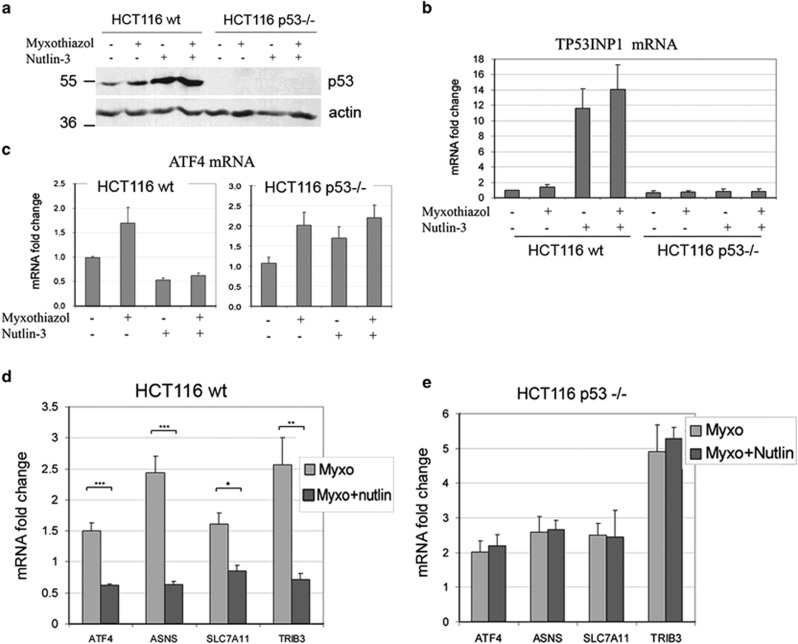
Preliminary p53 activation prevents the upregulation of ATF4 and its target genes in response to short respiration chain inhibition. (**a**) Western analysis of p53 in Nutlin-3 (16 h)/myxothiazol (4 h) treated HCT116 wt and p53 −/− cells. (**b**–**c**) The effects of Nutlin-3 (16h) and/or myxothiazol (4 h) on TP53INT1 and ATF4 mRNA levels in HCT116 wt and p53 −/− cells were examined by RT-qPCR. Mean and S.D. are presented of three independent experiments. (**d**–**e**) The effects of myxothiazol (4 h) or Nutlin-3 (16 h) and myxothiazol (4 h) on ATF4, ASNS, SLC7A11 and TRIB3 mRNA levels were examined by RT-qPCR in HCT116 wt cells (**d**) and HCT116 p53 −/− cells (**e**). Mean and S.D. are presented of three independent experiments. Student's *t*-test was used to analyze statistical significance (*P<0.05, **P<0.01, ***P< 0.001). All values are normalized to the level of the corresponding mRNA in the control (untreated) cells

To test directly whether the activation of p53 is responsible for the switch from the ATF4 mRNA upregulation to its downregulation during the sustained inhibition of complex III, we pre-activated p53 with the Mdm2 antagonist Nutlin-3, treated the cells with myxothiazol for 4 h and monitored by RT-qPCR the mRNA levels for ATF4, several ATF4-responsive genes and the p53-inducible gene TP53INP1. Nutlin-3 is known to stabilize p53 and activate transcription of p53 target genes.^11^ Indeed, we observed a substantial accumulation of p53 in response to Nutlin-3, an induction of TP53INP1 transcripts (Figures 5a and b) and a twofold decrease in ATF4 mRNA levels (Figure 5c). Although after a short treatment with myxothiazol (before p53 activation) the expression of ATF4 and its target genes ASNS, TRIB3 and SLC7A11 was increased, it was either reduced (ATF4, ASNS) or unchanged (TRIB3, SLC7A11) if the cells were pre-treated with Nutlin-3 (Figures 5c and d, Supplementary Figure S5 a–e). The difference seems to be due to p53-activation, because in the p53 knockout HCT116 cells treated with myxothiazol, the expression of ATF4 and its target genes was increased independent of the Nutlin-3 pretreatment (Figure 5e, Supplementary Figure S5 a–e). We conclude that p53 activation prevents the induction of ATF4 by ETC complex III inhibition.

### The set of ATF4 target genes upregulated by ETC complex III inhibition is observed only in the absence of p53 activation

Our results indicate that the switch from upregulation to downregulation of ATF4-dependent transcription after a sustained inhibition of complex III can be prevented by a supplementation with uridine that abolishes the induction of p53. We decided to identify the set of genes with a similar regulation mode based on the mRNA-seq data. We selected 131 genes (Supplementary Table S1) that show upregulation after the treatment with myxothiazol for 5 h, or with myxothiazol plus uridine for 13 h (when p53 is not activated), but do not increase after the treatment with myxothiazol alone for 13 h (when p53 is induced), and performed a gene ontology enrichment analysis. We included to the list 34 additional genes whose expression was upregulated in all treated samples, but reliably decreased after the treatment for 13 h with myxothiazol with no uridine supplementation, in comparison to the expression of the same genes after treatment for 13 h with myxothiazol plus uridine (Supplementary Table S2). The functional clustering among the combined gene list has revealed enrichment with transcripts encoding proteins involved in the aminoacyl-tRNA biosynthesis (FDR 0.0789) and in the transmembrane amino acid transporter activity (FDR 0.0299) as well as the genes involved in regulation of biosynthetic processes. As it was shown recently by analysis of the ChIP-Seq data, the biological functions of the most significantly enriched ATF4 target genes involve protein biosynthesis (including aminoacyl-tRNA synthetases), amino acid transport and amino acid biosynthesis.^10^ By comparing the list of ATF4 target genes based on the published ChIP-seq analysis^10^ with the list of genes obtained in this work (165 genes, Supplementary Tables S1 and S2), we found that all six aminoacyl-tRNA synthetases and six of seven transmembrane amino acid transporters from our gene list correspond to the known ATF4 target genes. Altogether, besides the above-described 5 ATF4 target genes tested by RT-qPCR in our study, 30 additional previously identified ATF4 target genes were found to be upregulated by myxothiazol in the absence of p53 activation (Table 2). The results strongly argue for the repressor activity of p53 against the ATF4-dependent gene expression and provide the list of genes for a search of potential new ATF4 transcription targets.

### Anti-apoptotic role of ATF4 in the cells treated with ETC complex III inhibitor in the absence of p53 activation

We have shown previously that complex III inhibition triggers p53-dependent apoptosis.^8^ According to the mRNA-seq data, expression of dozens of pro-apoptotic genes was significantly upregulated in response to exposure of HCT116 cells to myxothiazol for 13–17h (Supplementary Table S3).

Blocking the p53 activation by uridine dramatically reduced the expression of pro-apoptotic genes including AIFM3, BBC3 (PUMA), BCL2L11, Casp3, Casp7, FAS, Lrdd (PIDD), PMAIP1 (NOXA) and TNFRSF10B (KILLER/DR5), which is consistent with the prevention of myxothiazol-induced apoptosis upon uridine supplementation.^12^

**Figure 6:**
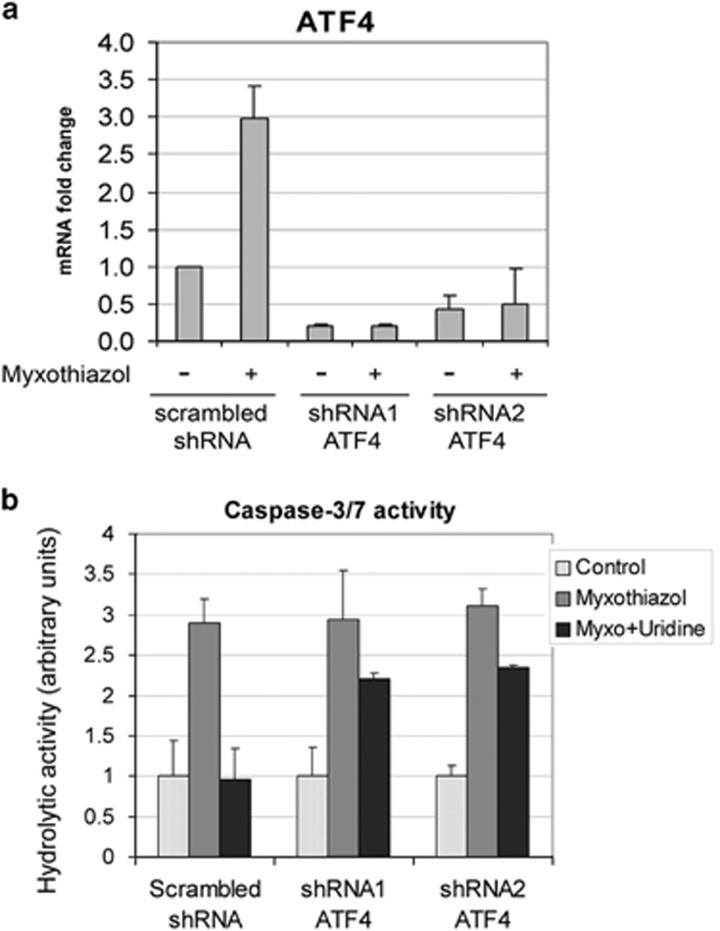
Uridine completely prevents myxothyazol-induced activation of caspases-3/7 in the control cells, but not in the cells with ATF4 knockdown. (**a**) RKO cells stably expressing either scrambled shRNA or ATF4 shRNA (shRNA1, shRNA2) were treated with 1 *μ*M myxothiazol for 3 h or left untreated. ATF4 mRNA levels were examined by RT-qPCR. (**b**) RKO cells stably expressing either scrambled shRNA or ATF4 shRNA (shRNA1, shRNA2) were treated with 1 *μ*M myxothiazol or 1 *μ*M myxothiazol+1 mM uridine for 24 h or left untreated (Control). Caspase-3/7 activity was measured as a relative rate of Ac-DEVD-AFC (20 ∝M) hydrolysis expressed as relative fluorescence units per hour (RFU/h). Mean and S.D. are presented of three independent experiments; each value was normalized to the corresponding value for the control (untreated) cells

According to controversial reports, ATF4 can either improve cell survival^13^ or induce oxidative stress and cell death.^10^ To reveal the role of ATF4 in cell fate after complex III inhibition, we used RKO cells stably expressing ATF4-specific shRNAs. The cells were treated with myxothiazol for 24 h in the absence and in the presence of uridine, and the activity of effector caspases was measured in cell lysates. Treatment with myxothiazol did not induce ATF4 transcripts in these cells (Figure 6a). In agreement with our previous results,^8, 12^ the treatment induced apoptosis and led to approximately threefold increase in caspase-3/7 activity (Figure 6b). We used this value as a positive control. As expected,^12^ uridine completely prevented the activation of caspases-3/7 in the cells with normal ATF4 levels, but a substantial activity of caspase-3/7 (73–76% of the positive control) was observed in the cells with ATF4 knockdown (Figure 6b). We conclude that if p53 activation is prevented by uridine, ATF4 plays a pro-survival role and protects the cells from apoptosis induced by complex III inhibition.

## Discussion

At the early time points of mitochondrial ETC complex III inhibition by myxothiazol, we detected phosphorylation of eIF2*α* and the induction of ATF4 mRNA and ATF4-regulated transcripts indicating the engagement of the eIF2alpha-ATF4 pathway. The results are in agreement with the published data showing that a mitochondrial dysfunction correlates with the upregulated expression of the UPR/ISR genes.^2, 3, 4^ However, at later time points of myxothiazol treatment, the ATF4 expression switches from upregulation to suppression. The effect was observed in three different cell lines and was associated with a restoration of the control expression levels of several ATF4 target genes. We also found that unlike myxothiazol, the complex I inhibitor piericidine induced a long-term transcriptional activation of ATF4 and its target genes.

One of the most important differences between the effects of complex I and III inhibition is the activation of p53 tumor suppressor due to an impairment of the *de novo* pyrimidine biosynthesis by complex III inhibitors.^8^ Several arguments indicate that p53 activation is responsible for the switch from ATF4 mRNA upregulation to downregulation. First, the downregulation of ATF4 coincides with the p53 activation. Second, the abolishment of p53 activation by uridine supplementation prevents the inhibition of ATF4 mRNA expression at later time points of complex III inhibition. Third, pre-activation of p53 with Nutlin-3 results in the downregulation of ATF4 mRNA and prevents the induction of ATF4 mRNA in response to a short-term inhibition of complex III. Finally, the set of genes that show upregulation in response to complex III inhibition only in the absence of p53 activation is enriched with ATF4 target genes.

Certainly, an expression of some ATF4 target genes might be regulated by other transcription factors besides the ATF4. However, by inhibiting ATF4 with two shRNAs targeting different sites of the mRNA, we found that the induction of TRIB3, SLC7A11 and ASNS mRNAs in response to a short-term exposure to myxothiazol is highly dependent on ATF4. The major role of ATF4 in transactivation of these genes is consistent with the published data.^14, 15, 16^ The ATF4 pathway was also shown to be solely responsible for the UPR that induces transcription of CHAC1^17^ during the ER stress.

Meanwhile, the knockdown of ATF4 did not prevent the induction of DDIT3/CHOP mRNA during a short exposure to myxothiazol. Perhaps, besides the ATF4-dependent mechanisms, other signaling pathways might participate in the regulation of CHOP expression during the short-term inhibition of complex III. Indeed, it is well established that under ER stress, three different pathways (PERK/eIF2*α*/ATF4, IRE1/XBP1 and ATF6) cooperate in controlling the transcription of CHOP.^18^ A suppressive effect of p53 on the IRE1/XBP1 branch of the UPR has been recently reported, which particularly affects the XBP-1 promoter transactivation and alternative splicing.^19, 20^ Therefore, the negative regulation of CHOP expression by p53 observed here may be mediated in part by the suppressive action of p53 on the IRE1/XBP1 signaling pathway.

Currently available results suggest that p53 may suppress at least two signaling pathways engaged during the ER stress/UPR, namely, the IRE1/XBP1-mediated pathway^19, 20^ and the ATF4-mediated pathway (results of the present study). In support of the high biological relevance of this mechanism, it was reported that genetic ablation of p53 sensitizes mice to induction of ER stress, whereas p53 is protective against the hepatotoxic effects of chronic ER stress.^19^

ATF4 is associated not only with the PERK/eIF2*α*/ATF4 pathway of UPR/ER stress. ATF4 is the common downstream target integrating signals from different eIF2 kinases that may mediate cellular responses to variety of stresses. Therefore, the described negative regulation of ATF4 by p53 represents a more general significance beyond the UPR.

The list of genes upregulated by the complex III inhibition in the absence of p53 activation is enriched with ATF4 target genes, in particular with aminoacyl-tRNA synthetases and transmembrane amino acid transporters. It suggests that this kind of mitochondrial dysfunction may be associated with amino acid deficiency. Mitochondrial ETC inhibition results in a shortage of energy (ATP), activation of glycolysis and glucose deficiency. Cancer cells may use amino acids as an alternative energy source under glucose deprivation.^21^ The reduced amino acid pools were shown to lead to protein kinase GCN2 activation, eIF2 phosphorylation and ATF4 induction to compensate the amino acid deficiency. The activation of GCN2-ATF4-ASNS pathway promotes the survival of cancer cells under nutrient deprivation.^21^ We suggest that the GCN2-eIF2*α*−ATF4 pathway may be also activated in response to complex III inhibition. The assumption is consistent with the report showing that the GCN2-ATF4 pathway is induced by the ATP-synthase (complex V) inhibitor oligomycin.^22^ However, signals engaged in the upregulation of ATF4 mRNA in response to ETC inhibition are yet to be identified.

It was shown recently that ATF4 might suppress p53 by a downregulation of p19ARF transcription.^23^ Here, we reveal a feedback mechanism that involves a suppression of ATF4 by p53. Therefore, p53 and ATF4 may function as antagonistic transcription factors, and perhaps the gene expression programs controlled by these factors might as well be mutually exclusive.

We present here an example of such antagonistic relations that apparently perform a switch from a pro-survival ATF4-mediated effects induced by a transient deficiency in the respiratory chain to a pro-apoptotic p53-mediated effects induced by a sustained inhibition of complex III. Besides the known pro-apoptotic activities of p53, the mechanism includes the shutdown of ATF4 transcription. This switch of gene expression programs can be prevented by uridine supplementation that abolishes the induction of p53, restores the ATF4-mediated gene expression and enforces the ATF4-dependent long-term cell survival (Figure 6). Therefore, the cross-talk between ATF4 and p53 plays an important role in deciding cell fate in response to mitochondrial dysfunction. Apparently, energy deficiency *per se* that occurs following an inhibition of respiratory chain is not recognized as a grave hazard. To help the cell to recover from the stress, the ATF4 survival mechanisms are being induced. However, a specific inhibition of complex III that is directly involved in *de novo* biosynthesis of pyrimidines represents a real hazard for genetic stability. Therefore, upon depletion of pyrimidine nucleotides pools, the p53 tumor suppressor jumps in and induces suicidal programs that involve not only different branches of the p53-dependent pro-apoptotic programs but also the shut-off of pro-survival activities of ATF4.

## Materials and Methods

### Cell lines and chemicals

The human carcinoma cell lines bearing wild-type p53—HCT116, RKO and HeLa—were grown in DMEM supplemented with 10% fetal calf serum (Thermo Scientific/HyClone, Logan, UT, USA). For mRNA-seq analysis, 50–60% confluent HCT116 cells were treated with 1 *μ*M mitochondrial ETC complex III inhibitor myxothiazol (Sigma-Aldrich Inc., St. Louis, MO, USA) for 5, 13, 17 h or with 1 *μ*M myxothiazol and 1 mM uridine for 13 h. For RT- qPCR analysis, 50–60% confluent cells were treated with 1 *μ*M myxothiazol or/and 1 mM uridine or 2 *μ*M complex I inhibitor piericidine (Sigma-Aldrich Inc.) for indicated periods of time (as described in the figure legends). The inhibitors were added in concentrations necessary to completely block respiration of HeLa cells. For activation of p53, the cells were treated with 10 *μ*M nutlin-3 (AdooQ BioScience, Irvine, CA, USA) for 12 h and then myxothiazol was added for 4 h to 1 *μ*M final concentration.

### mRNA seq

All RNA-seq experiments were performed in two biological replicates. RNA was extracted using TRIzol reagent (Invitrogen). For each sample, the quality of RNA was checked by capillary electrophoresis using Agilent 2100 Bioanalyzer. All samples had RIN>9. Library preparation was performed using TruSeq RNA sample prep kit v. 2 (Illumina, San Diego, CA, USA) following the manufacturer's protocol. Before sequencing, library concentration was assessed using Qubit fluorimeter (Invitrogen, Madison, WI, USA) and real-time PCR (primers: I-qPCR-1.1 AATGATACGGCGACCACCGAGAT and I-qPCR-2.1 CAAGCAGAAGACGGCATACGA).

Libraries were diluted to 11 pM and sequenced on Illumina HiSeq2000 instrument with 50 bp read length. Quality control and trimming were performed using CLC Genomics Workbench 6.5.1. High-quality reads were mapped on reference human genome GRCh37 using RNA-seq algorithm (mapping parameters—maximum two mismatches—only uniquely mapped reads allowed). Total gene reads were considered as a measure of gene expression level. To estimate the variation between replicates, we used Pearson correlation; for each experimental condition, square of the Pearson correlation coefficient was >0.9. For analysis of differential gene expression, R package “DESeq” was used.^24^ To determine significant differential expressed genes, *P*-value with false discovery rate (FDR) correction for multiple testing of 0.05 was used. DAVID gene functional annotation tool^9^ was used to identify enrichment with Gene Ontology term and other (KEGG pathways, key words, OMIM diseases pathways). FDR of 0.1 was used to identify significantly enriched terms. Raw sequencing data were deposited in NCBI under Bioproject accession number SRP043021.

### Antibodies and western analysis

For western analysis, cells were lysed in reporter lysis buffer (Promega Inc., Madison, WI, USA). Equivalent amounts of total protein were subjected to 12% SDS-polyacrylamide gel electrophoresis and processed as previously described.^25^ For phospho-eIF2*α* (Ser51) analysis, cells were harvested, washed with PBS and immediately lysed by boiling in SDS sample buffer for 5 min. Equivalent aliquots of cell lysates were subjected to 15% SDS-polyacrylamide gel electrophoresis and processed according to the phospho-eIF2*α* (Ser51) antibody manufacturer's protocol. Western blots were developed using sheep anti-mouse IgG or goat anti-rabbit IgG conjugated with horseradish peroxidase (GE Healthcare, Milwaukee, WI, USA) and Western Lightning Chemiluminescense Reagent (Life Sciences and Technology, Perkin-Elmer, Hopkinton, MA, USA).

Antibodies to p53 (DO-1), eIF2*α* (FL315) and actin (C-2) were from Santa Cruz Biotechnology Inc. Santa Cruz, CA, USA; antibodies to phospho-eIF2*α* (Ser51) were from Enzo Life Sciences Inc., Farmingdale, NY, USA.

### RNA isolation and real time PCR

Cells were subjected to the indicated stress conditions, total cellular RNA was isolated and treated with DNase I (Thermo Scientific/Pierce, Rockford, IL, USA), and cDNAs were synthesized by annealing 5 *μ*g of denatured total RNA with 0.2 *μ*g of random hexamers. The mixture was then incubated with 200 units of RevertAid Premium Reverse Transcriptase (Fermentas/Thermo Scientific, Waltham, MA, USA) for 10 min at 20 °C and for 60 min at 42 °C.

The qRT-PCR was performed using the CFX96 Real-time PCR detection system (Bio-Rad, USA) and the following primers: ATF4 dir CTTCACCTTCTTACAACCTCTTC, ATF4 rev GTAGTCTGGCTTCCTATCTCC; 18S rRNA dir CGGACAGGATTGACAGATTG, 18S rRNA rev CAGAGTCTCGTTCGTTATCG; CHOP dir CCTGCTTCTCTGGCTTGG, CHOP rev CTTGGTCTTCCTCCTCTTCC; TRIB3 dir AGGGAAGAGGAGGGAGAC, TRIB3 rev TCTGGAAGGCACTGAAGG, CHAC1 dir CTTCTCCTCCACCAGTTC, CHAC1 rev AGTAGATAGACAGACAGACAG; SLC7A11 dir CGCAAGCACACTCCTCTAC, SLC7A11 rev GCATATCTGGGCATTTGTATCG; ASNS dir CCGAGGAGGAGAGTGAGAGG, ASNS rev TGGTGGCAGAGACAAGTAATAGG; TP53INP1 dir TCAGCAGAAGAAGAAGAAGAAGAG, TP53INP1 rev AGCAGGAATCACTTGTATCAGC.

For the detection of target genes, the EVA Green master mix (Syntol, Moscow, Russia) was used according to the manufacturer's instructions. The thermal profile for EVA Green qRT-PCR included an initial heat-denaturing step at 95 °C for 3 min and 45 cycles at 95 °C for 15 s, an annealing step for 15 s and 72 °C for 20 s coupled with fluorescence measurements. Following amplification, the melting curves of the PCR products were monitored to determine the specificity of amplification. Each sample was run in triplicate, and a non-template control was added to each run.

Quantification was carried out using the Bio-Rad CFX Manager 3.0 software. Quantification of the target genes was normalized using the reference 18 S rRNA to compensate for inter-PCR variations. Values are a representation of three independent experiments, with standard deviations as indicated. Statistical significance was calculated by using the two-tailed Student's *t test*.

### Preparation of cell lines with knocked-down expression of ATF4

Lentiviral vector pLSLP^26^ was used to insert shRNAs targeting ATF4 mRNA with the following sequences: sh1—gatccgGCCTAGGTCTCTTAGATGATTCACGTGAATCATCT AAGAGACCTAGGCtttttg, sh2 – gatccgGCCAAGCACTTCAAACCTCATCACGTGATGAG GTTTGAAGTGCTTGGCtttttg. To obtain recombinant lentivirus stocks, 100 mm cell culture plates with 293T cells were transfected with the corresponding lentiviral construct (3 *μ*g) mixed with the set of packaging plasmids pRev2 (12 *μ*g), pGag1 (6 *μ*g) and pVSV-G (3 *μ*g)^26^ using LipofectAMIN 2000 (Invitrogen, Carlsbad, CA, USA). The next day, the medium was changed to 10 ml of DMEM, containing 2% FBS. Two days after transfection, culture medium containing viral particles was collected, filtered through a low protein binding membrane (0.45 *μ*M pore size) and stored at −80 °C.

RKO cells were infected with viral stocks encoding two different variants of ATF4 shRNAs (sh1ATF4 and sh2ATF4), or control viruses with scrambled shRNAs. For this purpose, 1 ml of viral stock diluted with 1 ml of fresh medium and 5–8 *μ*g/ml polybrene (hexadimethrine bromide, Sigma-Aldrich Inc.) were added to the cells on a 35 mm dish. Puromycin (1 *μ*g/ml) was added 3 days later and the selection of resistant cells was carried out for 5 days. Levels of ATF4 mRNA were determined by RT-qPCR.

### Assay of caspase-3/7 activity

Cells were treated under the indicated stress conditions, harvested, washed with PBS and lysed in 20 mm Tris-HCl (pH 7.5), 120 mM NaCl, 1 mM EDTA, 0.5%NP40. Lysates were precleared by centrifugation, protein concentrations were measured by Bradford assay, and the samples (20 *μ*g of total protein) were subjected to caspase-3/7 assay in 20 mM HEPES (pH 7.5), 50 mM NaCl, 5% glycerol, 0.1% Tween-20, 10 mM DTT and 20 *μ*M fluorogenic peptide substrate Ac-DEVD-AFC (American Peptides, Sunnyvale, CA, USA). Kinetic measurements of fluorescence were performed at 32 °C for 2 h in triplicate using FLUOstar OPTIMA reader equipped with 405 nm excitation and 520 nm emission filters (BMG Labtech, Offenburg, Germany), and Russian Government grant number 11.G34.31.0008 (M.D.L.).

## Figures and Tables

**Table 1:** Differential expression of ATF4 and its selected transcriptional targets in HCT116 cells after mitochondrial ETC complex III inhibition with myxothiazol for 5 h (M5), 13 h (M13), 17 h (M17) and 13 h in the presence of uridine (MU13)

*Gene ID*	*Gene name*	*mRNA fold changes*^a^			
		*M5*	*M13*	*M17*	*MU13*
Atf4	activating transcription factor 4 (tax- responsive enhancer element B67)	2.46	0.43	0.24	1.76
Trib3	tribbles homolog 3 (Drosophila)	5.41	NC	0.55	3.24
Ddit3	DNA-damage-inducible transcript 3, C/EBP homologous protein (CHOP)	5.53	2.07	NC	2.77
Chac1	ChaC, cation transport regulator homolog 1 (E. coli)	7,9	NC	0,28	4.98
Slc7a11	solute carrier family 7, (cationic amino acid transporter, y+ system) member 11	2.41	NC	0.24	4.76
Asns	asparagine synthetase	3.56	2.98	1.53	3.05

Abbreviation: NC, no change.

a Fold change in mRNA levels according to mRNA-seq data is presented (FDR<0.05).

**Table 2:** Differential expression of known transcriptional targets of ATF4 (except those listed in the Table 1) from the lists of genes upregulated by mitochondrial ETC complex III inhibition in the absence of p53 activation (presented in Tables S1 and S2). HCT116 cells were treated with myxothiazol for 5 h (M5), 13 h (M13), 17 h (M17) and for 13 h in the presence of uridine (MU13)

*#*	*Gene symbol*	*Ensemble ID*	a*mRNA fold changes*				*Ref*
			*M5*	*M13*	*M17*	*MU13*	
*Amino acid transporters*							
1	Slc7a11	ENSG00000151012	2.41	NC	0.24	4.76	
2	SLC7A1	ENSG00000139514	1.78	0.67	NC	1.94	
3	SLC6A9	ENSG00000196517	2.97	NC	NC	3.12	^10^
4	Slc38a2	ENSG00000134294	1.90	0.49	0.46	1.425	
5	SLC1A5	ENSG00000105281	1.69	1.27	NC	1.88	
6	SLC7A5	ENSG00000103257	1.81	1.89	NC	2.72	
							
*Aminoacyl-tRNA synthetases*							
7	AARS	ENSG00000090861	1.86	NC	0.60	1.98	
8	mars	ENSG00000166986	1.53	NC	NC	1.87	
9	SARS	ENSG00000031698	1.78	NC	0.77	2.10	^10^
10	Gars	ENSG00000106105	2.03	NC	0.71	1.62	
11	Yars	ENSG00000134684	1.62	NC	NC	1.71	
12	eprs	ENSG00000136628	1.43	NC	NC	1.29	
							
*Other ATF4 targets*							
13	FGF19	ENSG00000162344	1.97	NC	NC	1.97	^27^
14	ddit4	ENSG00000168209	4.36	NC	NC	1.95	
15	JDP2	ENSG00000140044	2.67	NC	NC	2.44	
16	CTH	ENSG00000116761	1.38	NC	NC	1.45	
17	Eif1	ENSG00000173812	2.06	0.66	NC	1.41	^10^
18	EIF4EBP1	ENSG00000187840	1.35	0.78	NC	1.41	
19	STC2	ENSG00000113739	2.63	NC	0.74	2.1	
20	GCLC	ENSG00000001084	1.72	NC	NC	1.51	^28^
21	Psat1	ENSG00000135069	2.5	NC	0.66	2.37	
22	ANK2	ENSG00000145362	1.93	NC	NC	2.06	
23	Klf4	ENSG00000136826	2.05	NC	0.66	1.45	
24	RHBDD1	ENSG00000144468	1.36	0.56	NC	1,41	
25	Xpot	ENSG00000184575	1.52	NC	0.64	1.55	^10^
26	FAM129A	ENSG00000135842	2.59	1.95	1.4	3.37	
27	GPT2	ENSG00000166123	1.59	1.34	NC	1.94	
28	Arhgef2	ENSG00000116584	1.83	1.58	NC	2.10	
29	Mid1ip1	ENSG00000165175	1.33	1.25	NC	1.72	
30	Gtpbp2	ENSG00000172432	2.15	1.30	NC	2.05	

Abbreviation: NC, no change.

a Fold change in mRNA levels according to mRNA-seq data is presented (FDR<0.05).

## Abbreviations

ASNS: asparagine synthetase
ATF: activating transcription factor
ChIP: chromatin immunoprecipitation
CHOP: C/EBP homologous protein
DAVID: Database for Annotation, Visualization and Integrated Discovery
eIF2*α*: eukaryotic initiation factor 2, *α* subunit
ER: endoplasmic reticulum
ETC: electron transfer chain
FDR: false discovery rate
GCN2: general control nondepressible 2
IRE1: inositol-requiring enzyme 1
ISR: integrated stress response
PERK: PKR-like endoplasmic reticulum kinase
qPCR: quantitative real-time PCR
RT: reverse transcription
shRNA: small hairpin RNA
UPR: unfolded protein response
XBP1: X-box binding protein 1
