# Supplementary material for: A sustained deficiency of mitochondrial respiratory complex III induces an apoptotic cell death through the p53-mediated inhibition of pro-survival activities of the activating transcription factor 4
Source: Cell Death Dis. 2014 Nov 6;5(11):e1511–. doi: 10.1038/cddis.2014.469 (PMC4260727; doi:10.1038/cddis.2014.469)
Supplement: Supplementary Figures [file cddis2014469x1.pdf]

Supplementary figures

A sustained deficiency of mitochondrial respiratory complex III induces an apoptotic cell death through the p53-mediated inhibition of pro-survival activities of the Activating Transcription Factor 4

Alexandra G. Evstafieva, Alisa A. Garaeva, Anastasia A. Khutornenko, Anna V. Klepikova, [1,] Maria D. Logacheva, Alexey A. Penin, German E. Novakovsky, Irina E. Kovaleva, and Peter M. Chumakov.

Titles and legends to figures

Figure S1. The time-dependent effect of mitochondrial ETC complex III inhibition on the expression of transcription factor ATF4 and its target genes in RKO cells. (a-f) The effects of myxothiazol (1 μM) for indicated intervals of time on ATF4, ASNS, CHOP, TRIB3, SLC7A11 and TP53INP1 mRNA levels in RKO cells were examined by RT-qPCR. Control – untreated cells. Mean and S.D. are presented of three independent experiments. All values are normalized to the level of the corresponding mRNA in the control (untreated) cells. (g,h) Western analysis of p53 (g) and ATF4 (h) in myxothiazol treated RKO cells for indicated intervals of time.

Figure S2. The time-dependent effect of mitochondrial ETC complex III inhibition on the expression of transcription factor ATF4 and its target genes in HeLa cells. (a-d) The effects of myxothiazol (1 μM) for indicated intervals of time on ATF4, SLC7A11, CHAC1 and CHOP mRNA levels in HeLa cells were examined by RT-qPCR. Control – untreated cells. Mean and S.D. are presented of three independent experiments. All values are normalized to the level of the corresponding mRNA in the control (untreated) cells.

Figure S3. Induction of TRIB3, ASNS and SLC7A11 expression in response to short exposure to myxothiazol strongly depends on ATF4. RKO cells stably expressing either scrambled shRNA (shRNA cntr) or ATF4 shRNA (shRNA1, shRNA2) were treated with 1 μM myxothiazol for 3h or left untreated. ATF4, TRIB3, SLC7A11 and ASNS mRNA levels were

examined by RT-qPCR. Mean and S.D. are presented of three independent experiments. All values are normalized to the level of the corresponding mRNA in the control untreated cells.

Figure S4. Abolishment of p53 activation by uridine supplementation prevents down-regulation of ATF4 in response to complex III inhibition and stimulates expression of ATF4-target genes. (a) p53 accumulation was analyzed by Western analysis in RKO cells treated for 13 h with1 μM myxothiazol or/and 1 mM uridine as indicated. (b-g) TP53INT1, ATF4, TRIB3, ASNS and SLC7A11 mRNA levels in RKO cells treated in the same way were examined by RT-qPCR. All values are normalized to the level of the corresponding mRNA in the control (untreated) cells. Mean and S.D. are presented of three independent experiments.

Figure S5. Preliminary p53 activation prevents up-regulation of ATF4 and its target genes in response to short respiration chain inhibition. (a-e) The effects of 1 μM myxothiazol (4h) or 10 μM Nutlin-3 (16 h) or Nutlin-3 (16h) and myxothiazol (4h) on ASNS, TRIB3, CHOP, CHAC1 and SLC7A11 mRNA levels were examined by RT-qPCR in HCT116 wt and p53 -/- cells. All values are normalized to the level of the corresponding mRNA in the control (untreated) cells. Mean and S.D. are presented of three independent experiments.

Figure S1

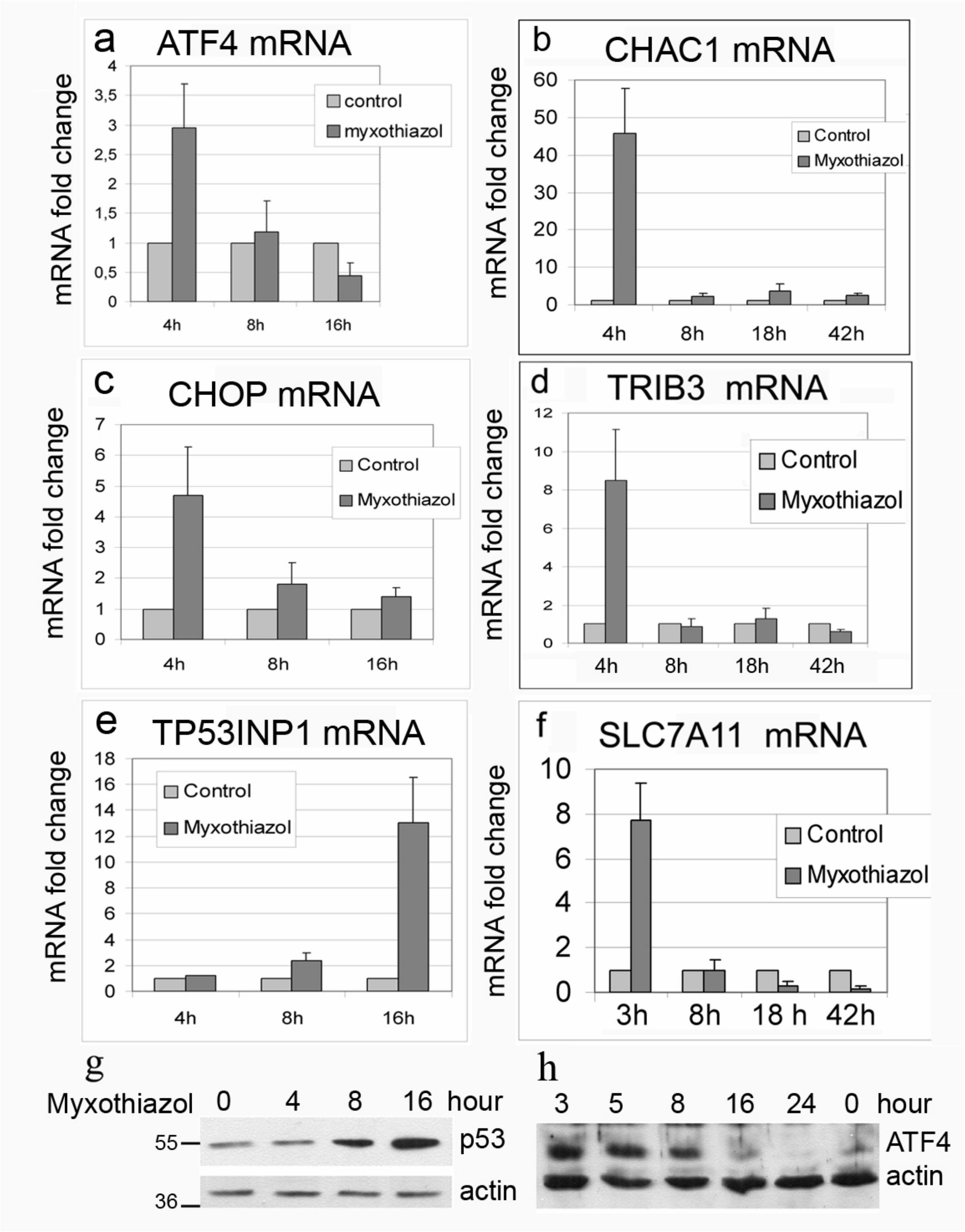
a ATF4 mRNA
control
myxothiazol
mRNA fold change
4h 8h 16h
b CHAC1 mRNA
Control
Myxothiazol
4h 8h 18h 42h
c CHOP mRNA
d TRIB3 mRNA
e TP53INP1 mRNA
f SLC7A11 mRNA
3h 8h 18 h 42h
g
Myxothiazol 0 4 8 16 hour
55
36
p53
actin
h
3 5 8 16 24 0 hour
ATF4
actin

Figure S2

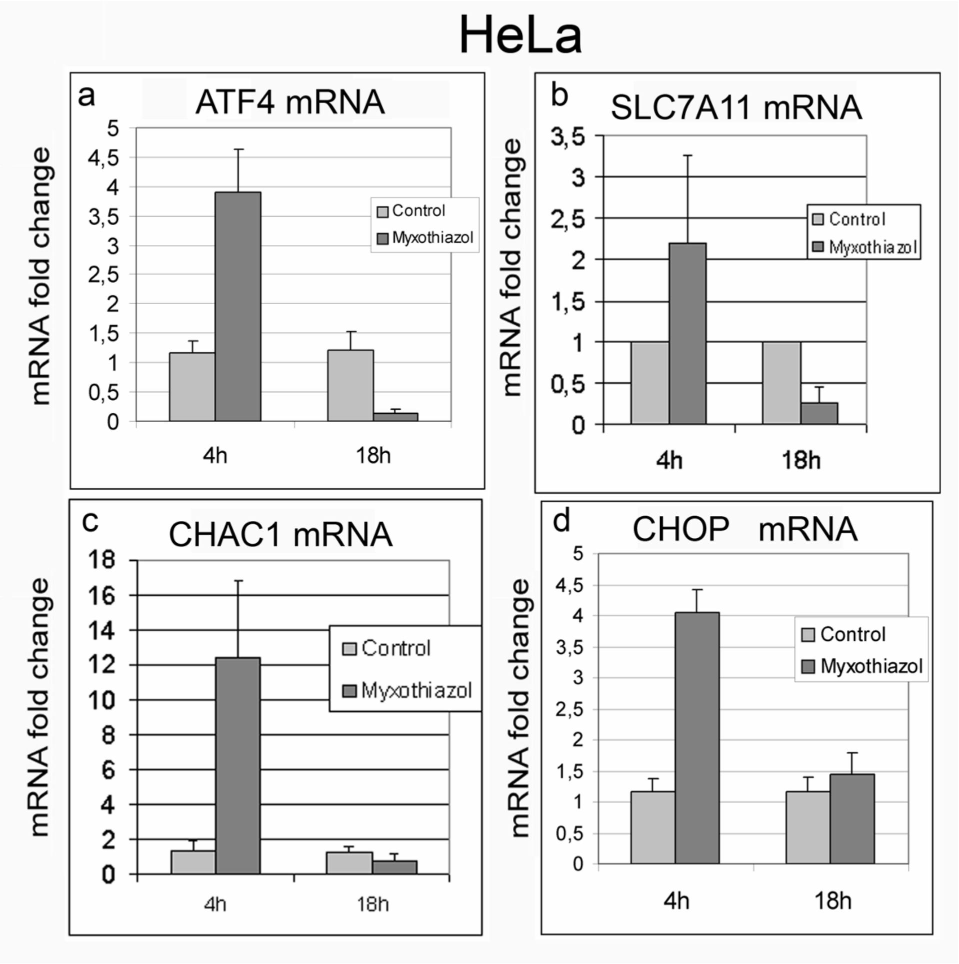

HeLa
a
ATF4 mRNA
mRNA fold change
5
4,5
4
3,5
3
2,5
2
1,5
1
0,5
0
Control
Myxothiazol
4h
18h
b
SLC7A11 mRNA
mRNA fold change
3,5
3
2,5
2
1,5
1
0,5
0
Control
Myxothiazol
4h
18h
c
CHAC1 mRNA
mRNA fold change
18
16
14
12
10
8
6
4
2
0
Control
Myxothiazol
4h
18h
d
CHOP mRNA
mRNA fold change
5
4,5
4
3,5
3
2,5
2
1,5
1
0,5
0
Control
Myxothiazol
4h
18h

Figure S3

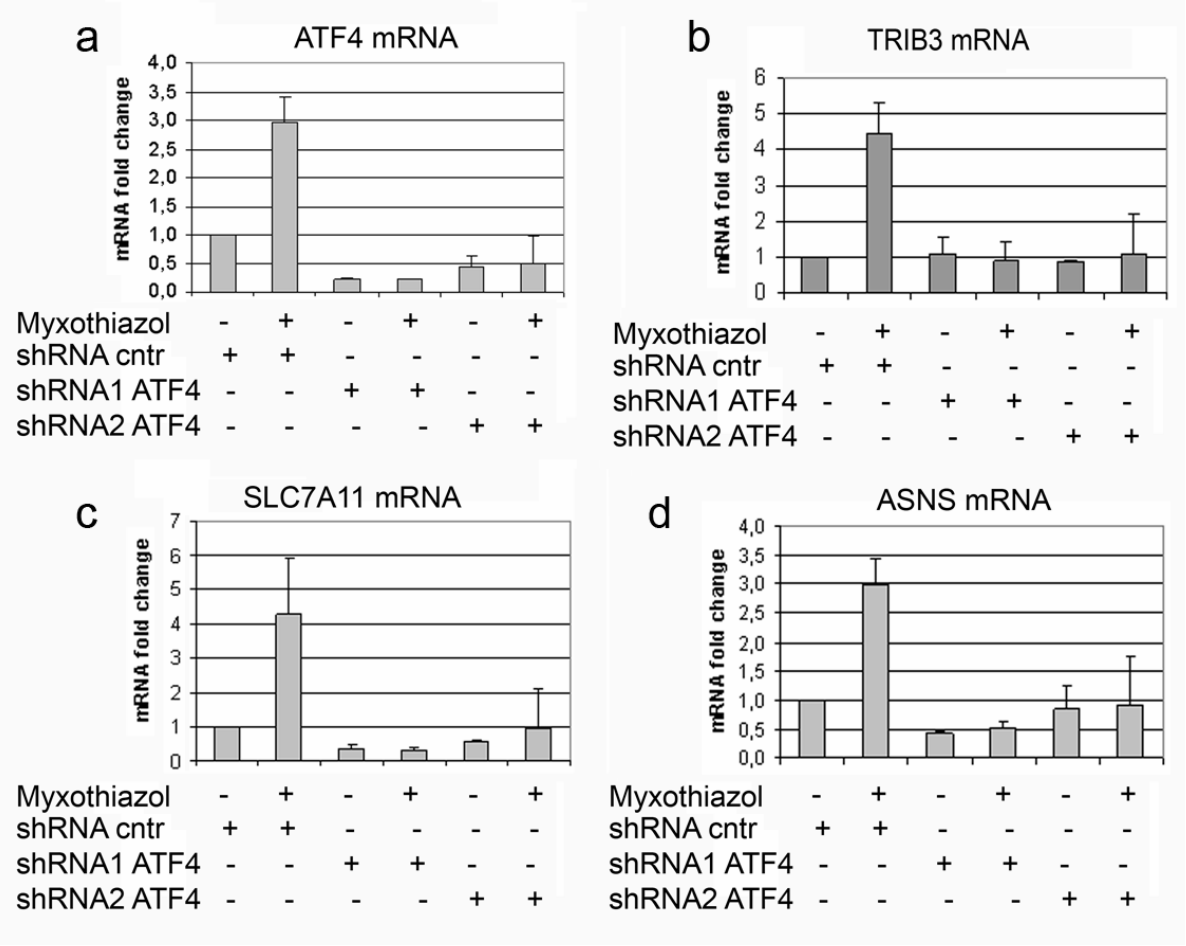
a
ATF4 mRNA
mRNA fold change
4,0
3,5
3,0
2,5
2,0
1,5
1,0
0,5
0,0
Myxothiazol - + - + - +
shRNA cntr + + - - - -
shRNA1 ATF4 - - + + - -
shRNA2 ATF4 - - - - + +
b
TRIB3 mRNA
mRNA fold change
6
5
4
3
2
1
0
Myxothiazol - + - + - +
shRNA cntr + + - - - -
shRNA1 ATF4 - - + + - -
shRNA2 ATF4 - - - - + +
c
SLC7A11 mRNA
mRNA fold change
7
6
5
4
3
2
1
0
Myxothiazol - + - + - +
shRNA cntr + + - - - -
shRNA1 ATF4 - - + + - -
shRNA2 ATF4 - - - - + +
d
ASNS mRNA
mRNA fold change
4,0
3,5
3,0
2,5
2,0
1,5
1,0
0,5
0,0
Myxothiazol - + - + - +
shRNA cntr + + - - - -
shRNA1 ATF4 - - + + - -
shRNA2 ATF4 - - - - + +

Figure S4

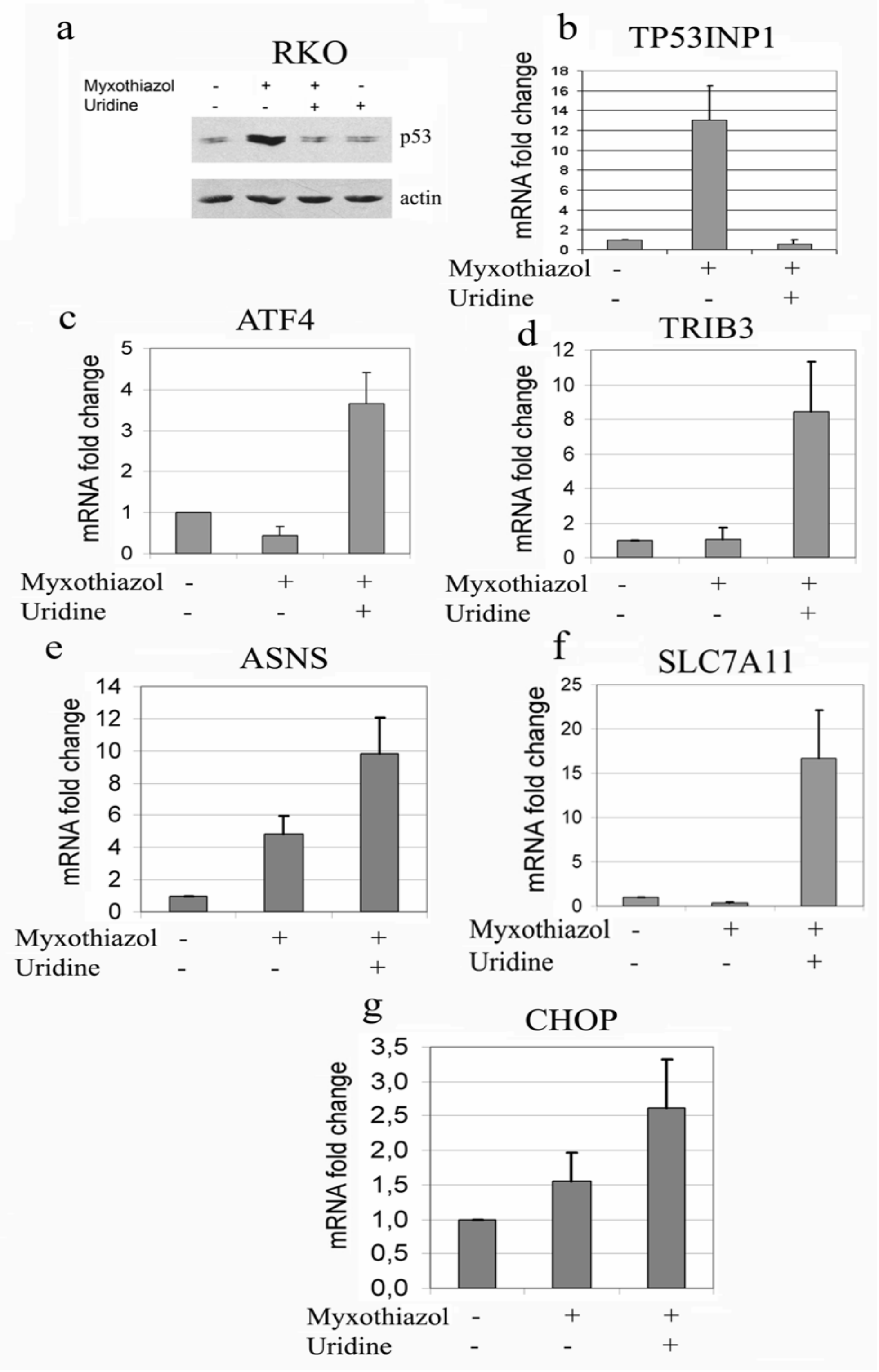
a
RKO
Myxothiazol - + + -
Uridine - - + +
p53
actin
b
TP53INP1
mRNA fold change
Myxothiazol - + +
Uridine - - +
c
ATF4
d
TRIB3
e
ASNS
f
SLC7A11
g
CHOP

Figure S5

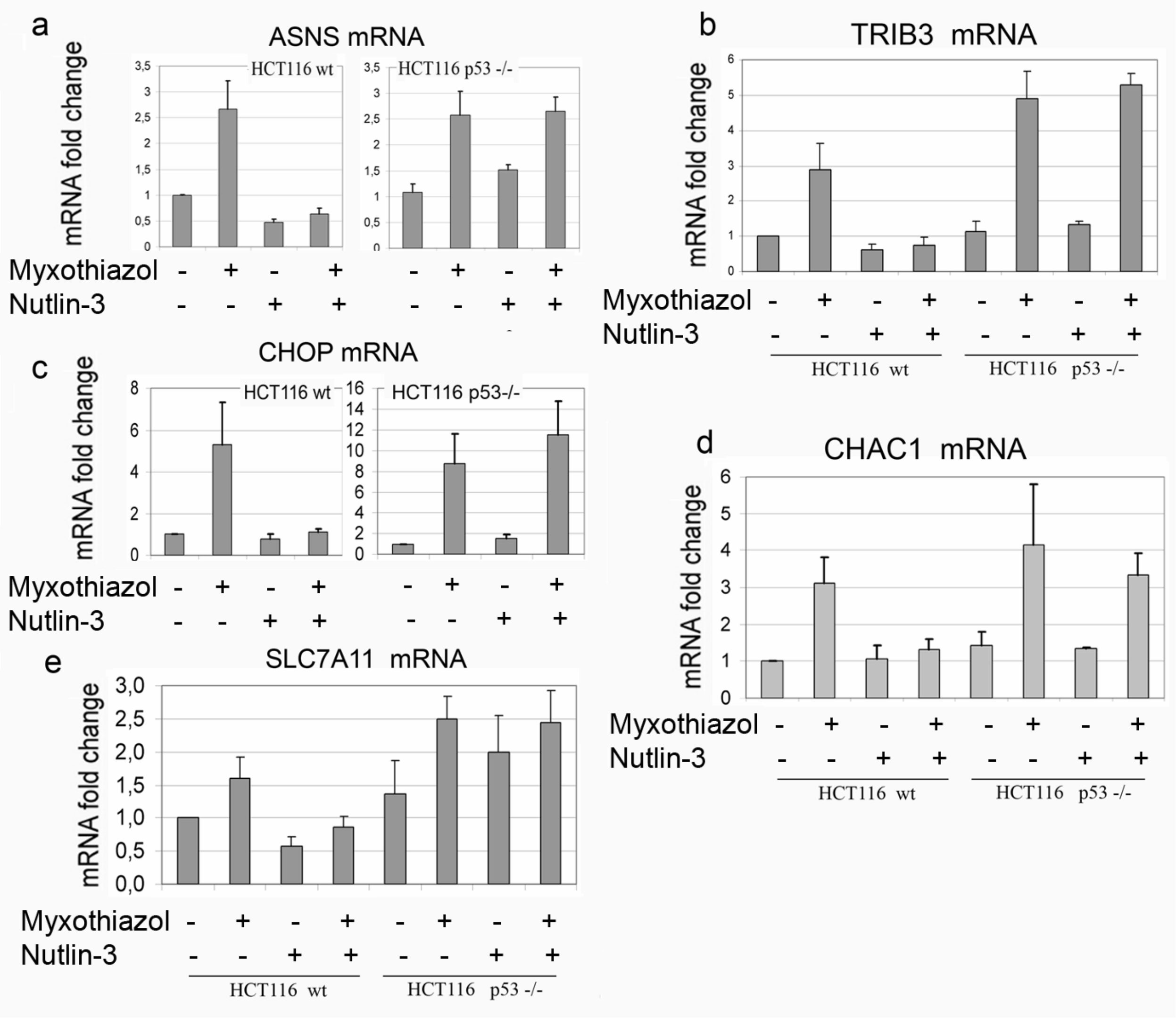

a
ASNS mRNA
mRNA fold change
HCT116 wt
HCT116 p53 -/-
Myxothiazol - + - + - + - +
Nutlin-3 - - + + - - + +
b
TRIB3 mRNA
mRNA fold change
Myxothiazol - + - + - + - +
Nutlin-3 - - + + - - + +
HCT116 wt
HCT116 p53 -/-
c
CHOP mRNA
mRNA fold change
HCT116 wt
HCT116 p53-/-
Myxothiazol - + - + - + - +
Nutlin-3 - - + + - - + +
d
CHAC1 mRNA
mRNA fold change
Myxothiazol - + - + - + - +
Nutlin-3 - - + + - - + +
HCT116 wt
HCT116 p53 -/-
e
SLC7A11 mRNA
mRNA fold change
Myxothiazol - + - + - + - +
Nutlin-3 - - + + - - + +
HCT116 wt
HCT116 p53 -/-
