# Supplementary material for: A sustained deficiency of mitochondrial respiratory complex III induces an apoptotic cell death through the p53-mediated inhibition of pro-survival activities of the activating transcription factor 4
Source: Cell Death Dis. 2014 Nov 6;5(11):e1511–. doi: 10.1038/cddis.2014.469 (PMC4260727; doi:10.1038/cddis.2014.469)
Supplement: Supplementary Tables [file cddis2014469x2.pdf]

Supplementary tables

A sustained deficiency of mitochondrial respiratory complex III induces an apoptotic cell death through the p53-mediated inhibition of pro-survival activities of the Activating Transcription Factor 4

Alexandra G. Evstafieva, Alisa A. Garaeva, Anastasia A. Khutornenko, Anna V. Klepikova, Maria D. Logacheva, Alexey A. Penin, German E. Novakovsky, Irina E. Kovaleva, and Peter M. Chumakov.

Table S1 A list of genes up-regulated by myxothiazol in the absence of p53 activation

A list of genes, up-regulated after treatment HCT116 cells with myxothiazol for 5 h or with myxothiazol and uridine for 13 h (no p53 activation), but not up-regulated after treatment with myxothiazol alone for 13 h (after p53 activation); mRNA-Seq results.

Ensemble ID	Gene symbol	David Gene Name
ENSG00000090861	AARS	alanyl-tRNA synthetase
ENSG00000128165	Adm2	adrenomedullin 2
ENSG00000026652	AGPAT4	1-acylglycerol-3-phosphate O-acyltransferase 4 (lysophosphatidic acid acyltransferase, delta)
ENSG00000131016	AKAP12	A kinase (PRKA) anchor protein 12
ENSG00000151743	AMN1	antagonist of mitotic exit network 1 homolog (S. cerevisiae)
ENSG00000145362	ank2	ankyrin 2, neuronal
ENSG00000138660	AP1AR	chromosome 4 open reading frame 16
ENSG00000172901	AQPEP	laeverin
ENSG00000109321	AREG	amphiregulin; amphiregulin B
ENSG00000124143	ARHGAP40	chromosome 20 open reading frame 95
ENSG00000117407	Artn	artemin
ENSG00000151693	Asap2	ArfGAP with SH3 domain, ankyrin repeat and PH domain 2
ENSG00000128272	ATF4	activating transcription factor 4 (tax-responsive enhancer element B67); activating transcription factor 4C
ENSG00000174718	C12orf35	chromosome 12 open reading frame 35
ENSG00000166780	c16orf45	chromosome 16 open reading frame 45
ENSG00000174749	c4orf32	chromosome 4 open reading frame 32
ENSG00000151881	C5orf28	chromosome 5 open reading frame 28
ENSG00000137038	C9orf123	chromosome 9 open reading frame 123
ENSG00000110888	CAPRIN2	caprin family member 2
ENSG00000105971	CAV2	caveolin 2
ENSG00000114654	CCDC48	coiled-coil domain containing 48
ENSG00000163814	CDCP1	CUB domain containing protein 1
ENSG00000168944	CEP120	centrosomal protein 120kDa
ENSG00000128965	CHAC1	ChaC, cation transport regulator homolog 1 (E. coli)
ENSG00000115295	CLIP4	CAP-GLY domain containing linker protein family, member 4
ENSG00000103647	CORO2B	coronin, actin binding protein, 2B
ENSG00000116761	CTH	cystathionase (cystathionine gamma-lyase)
ENSG00000168209	ddit4	DNA-damage-inducible transcript 4
ENSG00000142197	Dopey2	dopey family member 2
ENSG00000139318	dusp6	dual specificity phosphatase 6

ENSG00000107404	DVL1	dishevelled, dsh homolog 1 (Drosophila); dishevelled, dsh homolog 1 (Drosophila)-like 1
ENSG00000146648	egfr	epidermal growth factor receptor (erythroblastic leukemia viral (v-erb-b) oncogene homolog, avian)
ENSG00000120738	Egr1	early growth response 1
ENSG00000156976	EIF4A2	similar to eukaryotic translation initiation factor 4A2; eukaryotic translation initiation factor 4A, isoform 2
ENSG00000187840	EIF4EBP1	eukaryotic translation initiation factor 4E binding protein 1
ENSG00000134531	emp1	epithelial membrane protein 1
ENSG00000136628	eprs	glutamyl-prolyl-tRNA synthetase
ENSG00000124882	ereg	epiregulin
ENSG00000116285	ERRFI1	ERBB receptor feedback inhibitor 1
ENSG00000179715	FAM113B	family with sequence similarity 113, member B
ENSG00000101311	FERMT1	fermitin family homolog 1 (Drosophila)
ENSG00000162344	FGF19	fibroblast growth factor 19
ENSG00000166206	GABRB3	gamma-aminobutyric acid (GABA) A receptor, beta 3
ENSG00000106105	Gars	glycyl-tRNA synthetase
ENSG00000159131	GART	phosphoribosylglycinamide formyltransferase, phosphoribosylglycinamide synthetase, phosphoribosylaminoimidazole synthetase
ENSG00000001084	GCLC	glutamate-cysteine ligase, catalytic subunit
ENSG00000198380	GFPT1	glutamine-fructose-6-phosphate transaminase 1
ENSG00000112699	Gmds	GDP-mannose 4,6-dehydratase
ENSG00000159592	GPBP1L1	GC-rich promoter binding protein 1-like 1
ENSG00000125772	GPCPD1	hypothetical protein KIAA1434
ENSG00000023171	Gramd1b	GRAM domain containing 1B
ENSG00000106070	GRB10	growth factor receptor-bound protein 10
ENSG00000068097	HEATR6	HEAT repeat containing 6
ENSG00000122557	herpud2	HERPUD family member 2
ENSG00000182952	HMGN4	high mobility group nucleosomal binding domain 4
ENSG00000115756	hpcal1	hippocalcin-like 1
ENSG00000002587	Hs3st1	heparan sulfate (glucosamine) 3-O-sulfotransferase 1
ENSG00000003147	Ica1	islet cell autoantigen 1, 69kDa
ENSG00000160888	ier2	immediate early response 2
ENSG00000006652	ifrd1	interferon-related developmental regulator 1
ENSG00000164171	ITGA2	integrin, alpha 2 (CD49B, alpha 2 subunit of VLA-2 receptor)
ENSG00000008083	jarid2	jumonji, AT rich interactive domain 2
ENSG00000140044	JDP2	Jun dimerization protein 2
ENSG00000006459	Jhdm1d	jumonji C domain containing histone demethylase 1 homolog D (S. cerevisiae)
ENSG00000026559	KCNG1	potassium voltage-gated channel, subfamily G, member 1
ENSG00000104783	KCNN4	potassium intermediate/small conductance calcium-activated channel, subfamily N, member 4
ENSG00000114656	KIAA1257	KIAA1257
ENSG00000169926	KLF13	Kruppel-like factor 13
ENSG00000136826	Klf4	Kruppel-like factor 4 (gut)
ENSG00000102554	Klf5	Kruppel-like factor 5 (intestinal)
ENSG00000171401	KRT13	keratin 13
ENSG00000186007	LEMD1	LEM domain containing 1
ENSG00000124831	Lrrfip1	leucine rich repeat (in FLII) interacting protein 1
ENSG00000119681	LTBP2	latent transforming growth factor beta binding protein 2
ENSG00000166986	mars	methionyl-tRNA synthetase
ENSG00000112139	MDGA1	MAM domain containing glycosylphosphatidylinositol anchor 1
ENSG00000120254	MTHFD1L	methylenetetrahydrofolate dehydrogenase (NADP+ dependent) 1-like
ENSG00000065911	mthfd2	methylenetetrahydrofolate dehydrogenase (NADP+ dependent) 2, methenyltetrahydrofolate cyclohydrolase
ENSG00000188554	NBR1	neighbor of BRCA1 gene 1
ENSG00000135318	NT5E	5'-nucleotidase, ecto (CD73)
ENSG00000101188	Ntsr1	neurotensin receptor 1 (high affinity)
ENSG00000184232	OAF	OAF homolog (Drosophila)

ENSG00000205922	Onecut3	one cut homeobox 3
ENSG00000137819	Paqr5	progestin and adipoQ receptor family member V
ENSG00000168300	PCMTD1	protein-L-isoaspartate (D-aspartate) O-methyltransferase domain containing 1
ENSG00000150593	PDCD4	programmed cell death 4 (neoplastic transformation inhibitor)
ENSG00000060642	PIGV	phosphatidylinositol glycan anchor biosynthesis, class V
ENSG00000137193	Pim1	pim-1 oncogene
ENSG00000154217	PITPNC1	phosphatidylinositol transfer protein, cytoplasmic 1
ENSG00000010438	prss3	protease, serine, 3
ENSG00000135069	PSAT1	chromosome 8 open reading frame 62; phosphoserine aminotransferase 1
ENSG00000070159	Ptpn3	protein tyrosine phosphatase, non-receptor type 3
ENSG00000179912	R3HDM2	R3H domain containing 2
ENSG00000185989	Rasa3	RAS p21 protein activator 3
ENSG00000144468	RHBDD1	rhomboid domain containing 1
ENSG00000139725	Rhof	ras homolog gene family, member F (in filopodia)
ENSG00000013561	RNF14	ring finger protein 14
ENSG00000130363	RSPH3	radial spoke 3 homolog (Chlamydomonas)
ENSG00000159216	Runx1	runt-related transcription factor 1
ENSG00000031698	SARS	seryl-tRNA synthetase
ENSG00000064932	SBNO2	strawberry notch homolog 2 (Drosophila)
ENSG00000185033	SEMA4B	sema domain, immunoglobulin domain (Ig), transmembrane domain (TM) and short cytoplasmic domain, (semaphorin) 4B
ENSG00000147010	Sh3kbp1	SH3-domain kinase binding protein 1
ENSG00000125089	SH3TC1	SH3 domain and tetratricopeptide repeats 1
ENSG00000144136	SLC20A1	solute carrier family 20 (phosphate transporter), member 1
ENSG00000134294	slc38a2	solute carrier family 38, member 2
ENSG00000149150	SLC43A1	solute carrier family 43, member 1
ENSG00000196517	SLC6A9	solute carrier family 6 (neurotransmitter transporter, glycine), member 9
ENSG00000139514	SLC7A1	solute carrier family 7 (cationic amino acid transporter, y+ system), member 1
ENSG00000151012	SLC7A11	solute carrier family 7, (cationic amino acid transporter, y+ system) member 11
ENSG00000166949	SMAD3	SMAD family member 3
ENSG00000108854	SMURF2	SMAD specific E3 ubiquitin protein ligase 2
ENSG00000072310	Srebf1	sterol regulatory element binding transcription factor 1
ENSG00000113739	STC2	stanniocalcin 2
ENSG00000196182	Stk40	serine/threonine kinase 40
ENSG00000135269	tes	testis derived transcript (3 LIM domains)
ENSG00000150403	TMCO3	transmembrane and coiled-coil domains 3
ENSG00000067182	TNFRSF1A	tumor necrosis factor receptor superfamily, member 1A
ENSG00000101255	TRIB3	tribbles homolog 3 (Drosophila)
ENSG00000102804	Tsc22d1	TSC22 domain family, member 1
ENSG00000111981	ULBP1	UL16 binding protein 1
ENSG00000128564	VGF	VGF nerve growth factor inducible
ENSG00000100219	Xbp1	X-box binding protein 1
ENSG00000184575	Xpot	exportin, tRNA (nuclear export receptor for tRNAs); similar to Exportin-T (tRNA exportin) (Exportin(tRNA))
ENSG00000134684	Yars	tyrosyl-tRNA synthetase
ENSG00000188177	ZC3H6	zinc finger CCCH-type containing 6
ENSG00000104231	Zfand1	zinc finger, AN1-type domain 1
ENSG00000128016	ZFP36	zinc finger protein 36, C3H type, homolog (mouse)
ENSG00000185650	zfp36l1	zinc finger protein 36, C3H type-like 1
ENSG00000160961	ZNF333	zinc finger protein 333
ENSG00000198205	ZXDA	zinc finger, X-linked, duplicated A

Table S2 A list of genes whose expression was up-regulated in HCT116 cells in all three cases (myxothiazol 5h, 13h, myxothiazol +uridine 13h), but reliably decreased after myxothiazol alone treatment for 13 h in comparison to the expression of the same genes after treatment by myxothiazol and uridine for 13 h. mRNA-Seq results.

Ensemble ID	Gene symbol	David Gene Name
ENSG00000179833	SERTAD2	SERTA domain containing 2
ENSG00000184489	ptp4a3	protein tyrosine phosphatase type IVA, member 3
ENSG00000187720	THSD4	thrombospondin, type I, domain containing 4
ENSG00000101280	Angpt4	angiopoietin 4
ENSG00000205420	KRT6A	keratin 6A
ENSG00000163216	SPRR2D	small proline-rich protein 2D
ENSG00000136068	FLNB	filamin B, beta (actin binding protein 278)
ENSG00000175197	DDIT3	DNA-damage-inducible transcript 3
ENSG00000125826	RBCK1	RanBP-type and C3HC4-type zinc finger containing 1
ENSG00000120875	DUSP4	dual specificity phosphatase 4
ENSG00000185567	AHNAK2	AHNAK nucleoprotein 2
ENSG00000196352	Cd55	CD55 molecule, decay accelerating factor for complement (Cromer blood group)
ENSG00000138678	AGPAT9	1-acylglycerol-3-phosphate O-acyltransferase 9
ENSG00000005884	ITGA3	integrin, alpha 3 (antigen CD49C, alpha 3 subunit of VLA-3 receptor)
ENSG00000198431	TXNRD1	thioredoxin reductase 1; hypothetical LOC100130902
ENSG00000049239	H6pd	hexose-6-phosphate dehydrogenase (glucose 1-dehydrogenase)
ENSG00000166750	Slfn5	schlafen family member 5
ENSG00000166123	GPT2	glutamic pyruvate transaminase (alanine aminotransferase) 2
ENSG00000183696	upp1	uridine phosphorylase 1
ENSG00000175592	FOSL1	FOS-like antigen 1
ENSG00000135842	FAM129A	family with sequence similarity 129, member A
ENSG00000105281	SLC1A5	solute carrier family 1 (neutral amino acid transporter), member 5
ENSG00000167767	KRT80	keratin 80
ENSG00000132470	ITGB4	integrin, beta 4
ENSG00000137936	bcar3	breast cancer anti-estrogen resistance 3
ENSG00000103257	SLC7A5	solute carrier family 7 (cationic amino acid transporter, y+ system), member 5
ENSG00000116584	Arhgef2	Rho/Rac guanine nucleotide exchange factor (GEF) 2
ENSG00000165175	mid1ip1	MID1 interacting protein 1 (gastrulation specific G12 homolog (zebrafish))
ENSG00000186832	Krt16	keratin 16; keratin type 16-like
ENSG00000135480	Krt7	keratin 7
ENSG00000187678	SPRY4	sprouty homolog 4 (Drosophila)
ENSG00000169504	CLIC4	chloride intracellular channel 4
ENSG00000156510	HKDC1	hexokinase domain containing 1
ENSG00000172432	gtpbp2	GTP binding protein 2

Table S3. Differential expression of apoptotic genes in HCT116 cells on the basis of mRNA-Seq data after mitochondrial ETC complex III inhibition with myxothiazol for 5h (M5), 13 h (M13), 17 h (M17) and for 13 h in the presence of uridine (MU13). N16 – treatment with Nutlin-3 for 16 h. Fold changes in mRNA levels are presented (FDR < 0.05). NC – no change.

Ensemble ID	Gene symbol	David Gene Name	mRNA fold changes				
			M5	M13	M17	MU13	N16
ENSG00000143382	ADAMTSL4	ADAMTS-like 4	NC	3.0	3.2	NC	NC
ENSG00000183773	aifm3	apoptosis-inducing factor, mitochondrion-associated, 3	NC	4.01	3.2	NC	NC
ENSG00000105290	Aplp1	amyloid beta (A4) precursor-like protein 1	NC	2.57	3.09	NC	NC
ENSG00000105327	BBC3	BCL2 binding component 3	1.45	1.89	2.96	NC	6.40
ENSG00000153094	BCL2L11	BCL2-like 11 (apoptosis facilitator)	NC	2.01	2.06	NC	NC
ENSG00000100290	BIK	BCL2-interacting killer (apoptosis-inducing)	1.88	1.38	2.04	NC	2.1
ENSG00000166619	BLCAP	bladder cancer associated protein	NC	2.47	2.47	NC	2.18
ENSG00000164305	Casp3	caspase 3, apoptosis-related cysteine peptidase	NC	2.44	2.57	NC	NC
ENSG00000165806	casp7	caspase 7, apoptosis-related cysteine peptidase	NC	1.68	2.76	0.67	NC
ENSG00000144655	CSRNP1	cysteine-serine-rich nuclear protein 1	1.59	3.01	4.13	NC	2.01
ENSG00000110925	CSRNP2	cysteine-serine-rich nuclear protein 2	1.66	4.29	3.74	NC	NC
ENSG00000136048	DRAM1	DNA-damage regulated autophagy modulator 1	NC	1.51	2.08	NC	2.98
ENSG00000026103	Fas	Fas (TNF receptor superfamily, member 6)	NC	2.50	3.40	NC	4.25
ENSG00000116717	GADD45A	growth arrest and DNA-damage-inducible, alpha	1.82	2.77	2.55	1.52	3.72
ENSG00000111653	Ing4	inhibitor of growth family, member 4	NC	2.37	2.41	NC	NC
ENSG00000177606	Jun	jun oncogene	1.68	3.26	4.91	NC	NC
ENSG00000177595	Lrdd	leucine-rich repeats and death domain containing	NC	2.47	2.97	NC	2.31
ENSG00000165943	Moap1	modulator of apoptosis 1	NC	3.10	3.08	NC	NC
ENSG00000064300	Ngfr	nerve growth factor receptor (TNFR superfamily, member 16)	2.99	3.52	4.18	NC	NC
ENSG00000091592	Nlrp1	NLR family, pyrin domain containing 1	2.60	2.15	2.36	1.73	7.54
ENSG00000181649	phlda2	pleckstrin homology-like domain, family A, member 2	2.07	2.68	3.65	NC	NC
ENSG00000174307	Phlda3	pleckstrin homology-like domain, family A, member 3	NC	1.58	2.80	0.73	4.88
ENSG00000141682	PMAIP1	phorbol-12-myristate-13-acetate-induced protein 1	2.04	4.79	3.10	1.72	NC
ENSG00000164543	STK17A	serine/threonine kinase 17a	1.37	1.95	2.48	NC	NC
ENSG00000120889	tnfrsf10b	tumor necrosis factor receptor superfamily, member 10b	1.60	2.77	2.42	1.30	2.74
ENSG00000125657	TNFSF9	tumor necrosis factor (ligand) superfamily, member 9	NC	3.34	3.23	NC	NC
ENSG00000164938	TP53INP1	tumor protein p53 inducible nuclear protein 1	NC	10.78	28.58	NC	125.4
